# Supplementary material for: ESBL-Positive Enterobacteriaceae from Dogs of Santiago and Boa Vista Islands, Cape Verde: A Public Health Concern
Source: Antibiotics (Basel). 2023 Feb 23;12(3):447. doi: 10.3390/antibiotics12030447 (PMC10044620; doi:10.3390/antibiotics12030447)
Supplement: Supplementary file 1 [file antibiotics-12-00447-s001.zip › Table S1.pdf]

Supplementary file - 1

Table S1. Sampling data.

Sample (No)	Sampling location	Date	Estimated age interval (years)	Sex	Spay/Neuter Status	Confinement status	BCS	Presence of external parasites	Shelter
1	Bons Amigos	11/09/21	< 1	F	No	C	2	No	No
2	Bons Amigos	11/09/21	< 0,5	M	No	C	3	No	No
3	Bons Amigos	11/09/21	> 5	M	No	C	3	No	No
4	Bons Amigos	11/09/21	< 0,5	M	No	C	3	Yes	No
5	Bons Amigos	13/09/21	> 5	M	No	C	3	Yes	No
6	Bons Amigos	13/09/21	< 5	F	No	NC	2	No	No
7	Bons Amigos	13/09/21	< 0,5	F	No	NC	3	Yes	No
8	Bons Amigos	13/09/21	> 5	M	No	C	3	Yes	No
9	Bons Amigos	16/09/21	> 5	M	No	NC	2	Yes	No
10	Bons Amigos	16/09/21	< 5	F	No	NC	2	Yes	No
11	Bons Amigos	17/09/21	< 5	M	No	NC	1	Yes	No
12	Bons Amigos	18/09/21	<5	M	No	C	3	Yes	No
13	Bons Amigos	18/09/21	>5	M	No	C	3	Yes	No
14	Bons Amigos	20/09/21	>5	F	No	C	4	Yes	No
15	Bons Amigos	20/09/21	<5	F	No	C	3	Yes	No
16	Bons Amigos	20/09/21	<5	F	No	C	2	Yes	No
17	Bons Amigos	22/09/21	>5	M	No	C	3	Yes	No
18	Bons Amigos	22/09/21	<5	F	No	C	2	Yes	No
19	Bons Amigos	22/09/21	>5	M	No	C	3	Yes	No

number (No); female (F); male (M); confined (C); not confined (ND); Body Condition Score (BCS).

Table S1. cont.

Sample (No)	Sampling location	Date	Estimated age interval (years)	Sex	Spay/Neuter Status	Confinement status	BCS	Presence of external parasites	Shelter
20	Bons Amigos	23/09/21	>5	F	No	NC	1	Yes	No
21	Bons Amigos	23/09/21	<5	F	No	NC	3	Yes	No
22	Bons Amigos	24/09/21	<5	F	No	NC	1	Yes	No
23	Bons Amigos	24/09/21	<5	F	No	C	3	Yes	No
24	Bons Amigos	24/09/21	<5	F	No	NC	1	Yes	No
25	Bons Amigos	24/09/21	<5	F	No	NC	3	Yes	No
26	Bons Amigos	25/09/21	<5	F	No	NC	2	Yes	No
27	Bons Amigos	25/09/21	<5	F	No	C	3	Yes	No
28	Bons Amigos	25/09/21	>5	F	No	C	3	Yes	No
29	Bons Amigos	25/09/21	<5	F	No	C	2	Yes	No
30	Bons Amigos	25/09/21	<5	F	No	C	2	Yes	No
31	Bons Amigos	27/09/21	<5	F	No	NC	2	Yes	No
32	Bons Amigos	27/09/21	<5	F	No	NC	2	Yes	No
33	Bons Amigos	27/09/21	<5	F	No	NC	2	Yes	No
34	Bons Amigos	27/09/21	<5	F	No	NC	2	Yes	No
35	Bons Amigos	27/09/21	<5	F	No	C	2	Yes	No
36	Bons Amigos	28/09/21	<1	M	No	C	2	Yes	No
37	Bons Amigos	28/09/21	<5	M	No	C	2	Yes	No
38	Bons Amigos	28/09/21	<5	M	No	NC	2	Yes	No
39	Bons Amigos	28/09/21	<5	M	No	NC	2	Yes	No
40	Bons Amigos	28/09/21	<5	F	No	NC	1	Yes	No

number (No); female (F); male (M); confined (C); not confined (ND); Body Condition Score (BCS).

Table S1. cont.

Sample (No)	Sampling location	Date	Estimated age interval (years)	Sex	Spay/Neuter Status	Confinement status	BCS	Presence of external parasites	Shelter
41	Bons Amigos	28/09/21	<5	F	No	C	2	Yes	No
42	Bons Amigos	29/09/21	<5	F	No	C	3	Yes	No
43	Bons Amigos	29/09/21	<5	M	No	NC	2	Yes	No
44	Bons Amigos	29/09/21	<5	M	No	C	3	Yes	No
45	Bons Amigos	29/09/21	<5	F	No	NC	3	Yes	No
46	Bons Amigos	29/09/21	<5	F	No	NC	3	Yes	No
47	Bons Amigos	29/09/21	<5	M	No	NC	2	Yes	No
48	Bons Amigos	29/09/21	>5	F	No	NC	4	Yes	No
49	Bons Amigos	29/09/21	>5	F	No	NC	1	Yes	No
50	Bons Amigos	29/09/21	<5	F	No	NC	2	Yes	No
51	Nerina	03/11/21	<5	F	Yes	C	3	No	Yes
52	Nerina	03/11/21	<5	F	Yes	C	3	No	Yes
53	Nerina	03/11/21	<5	F	Yes	C	3	No	Yes
54	Nerina	03/11/21	<5	F	Yes	C	3	No	Yes
55	Nerina	03/11/21	<5	M	Yes	C	3	No	Yes
56	Nerina	03/11/21	<5	F	Yes	C	3	No	Yes
57	Nerina	03/11/21	<5	M	Yes	C	3	No	Yes
58	Nerina	03/11/21	<5	F	Yes	C	3	No	Yes
59	Nerina	03/11/21	<5	F	Yes	C	3	No	Yes
60	Nerina	04/11/21	<5	F	Yes	C	3	No	Yes

number (No); female (F); male (M); confined (C); not confined (ND); Body Condition Score (BCS).

Table S1. cont.

Sample (No)	Sampling location	Date	Estimated age interval (years)	Sex	Spay/Neuter Status	Confinement status	BCS	Presence of external parasites	Shelter
61	Nerina	04/11/21	<5	M	Yes	C	3	No	Yes
62	Nerina	04/11/21	<5	M	Yes	C	3	No	Yes
63	Nerina	04/11/21	<5	M	Yes	C	3	No	Yes
64	Nerina	03/11/21	<5	M	Yes	NC	2	Yes	No
65	Nerina	03/11/21	<5	M	No	NC	4	Yes	No
66	Nerina	03/11/21	<1	M	No	NC	2	Yes	No
67	Nerina	04/11/21	<5	F	No	NC	3	No	No
68	Nerina	04/11/21	>5	M	Yes	NC	3	No	No
69	Nerina	04/11/21	<5	M	Yes	C	3	No	Yes
70	Nerina	04/11/21	<5	M	Yes	C	3	No	Yes
71	Nerina	05/11/21	<5	M	No	NC	2	No	No
72	Nerina	05/11/21	<5	M	No	NC	3	No	No
73	Nerina	05/11/21	<5	F	Yes	C	3	No	No
74	Nerina	08/11/21	<5	F	No	NC	3	No	No
75	Nerina	08/11/21	<5	F	Yes	C	2	Yes	No
76	Nerina	08/11/21	<5	F	Yes	C	3	No	No
77	Nerina	08/11/21	<5	F	Yes	NC	3	No	No
78	Nerina	08/11/21	>5	F	Yes	C	4	No	No
79	Nerina	09/11/21	<5	M	Yes	C	3	No	No
80	Nerina	09/11/21	<5	F	Yes	NC	3	No	No

number (No); female (F); male (M); confined (C); not confined (ND); Body Condition Score (BCS).

Table S1. cont.

Sample (No)	Sampling location	Date	Estimated age interval (years)	Sex	Spay/Neuter Status	Confinement status	BCS	Presence of external parasites	Shelter
81	Nerina	10/11/21	<5	F	Yes	NC	3	No	No
82	Nerina	11/11/21	<5	M	Yes	NC	3	No	No
83	Nerina	11/11/21	<5	F	Yes	NC	3	No	No
84	Nerina	15/11/21	<5	F	No	NC	3	No	No
85	Nerina	15/11/21	>5	M	Yes	NC	3	Yes	No
86	Nerina	17/11/21	<5	F	Yes	C	3	No	No
87	Nerina	18/11/21	<5	F	Yes	NC	3	No	No
88	Nerina	18/11/21	>5	F	Yes	NC	2	Yes	No
89	Nerina	19/11/21	<5	M	Yes	NC	1	No	No
90	Nerina	22/11/21	>5	M	Yes	C	3	Yes	No
91	Nerina	22/11/21	>5	M	Yes	NC	3	Yes	No
92	Nerina	23/11/21	<5	M	No	NC	2	Yes	No
93	Nerina	23/11/21	<5	M	No	NC	2	Yes	No
94	Nerina	25/11/21	<5	M	Yes	C	2	No	No
95	Nerina	25/11/21	<5	M	No	NC	2	No	No
96	Nerina	25/11/21	>5	M	No	NC	2	No	No
97	Nerina	26/11/21	<5	F	No	C	4	No	No
98	Nerina	26/11/21	<5	M	No	NC	2	Yes	No
99	Nerina	26/11/21	<5	M	No	NC	2	Yes	No
100	Nerina	30/11/21	<5	F	No	C	3	No	No

number (No); female (F); male (M); confined (C); not confined (ND); Body Condition Score (BCS).
