# Supplementary material for: ESBL-Positive Enterobacteriaceae from Dogs of Santiago and Boa Vista Islands, Cape Verde: A Public Health Concern
Source: Antibiotics (Basel). 2023 Feb 23;12(3):447. doi: 10.3390/antibiotics12030447 (PMC10044620; doi:10.3390/antibiotics12030447)
Supplement: Supplementary file 1 [file antibiotics-12-00447-s001.zip › Table S2.pdf]

Supplementary file – 2

Table S2. Results of the ESBL screening and confirmation, and identification protocols of the Enterobacteriaceae isolates.

Sample (No)	Isolate (No)	Colour on ChromID ESBL	Gram	Oxidase	MAC	Identification technique	ID	MDDST
1	1a	Pink to red	Neg	Neg	Neg	IMViC	*Morganella morganii*	Pos
5	5a	Pink to red	Neg	Neg	Pos	IMViC	*E.coli*	Pos
5	5b	Dark to light brown	Neg	Neg	Pos	IMViC	*E.coli*	Pos
5	5c	Bluish green to brownish	Neg	Neg	Pos	IMViC	*E.coli*	Pos
5	5d	Bluish green to brownish	Neg	Neg	Pos	IMViC	*E.coli*	Pos
5	5e	Bluish green to brownish	Neg	Neg	Neg	IMViC	*Proteus mirabilis*	Neg
5	5f	Bluish green to brownish	Neg	Neg	Neg	IMViC	*Proteus mirabilis*	Neg
10	10a	Bluish green to brownish	Neg	Neg	Pos	API 20E	*Citrobacter freundii*	Pos
10	10b	Bluish green to brownish	Neg	Neg	Pos	API 20E	*Citrobacter freundii*	Pos
10	10d	Bluish green to brownish	Neg	Neg	Pos	API 20E	*Citrobacter freundii*	Pos
13	13a	Pink to red	Neg	Neg	Neg	API 20E	*E.coli*	Pos
14	14a	Dark to light brown	Neg	Neg	Neg	API 20E	*Proteus vulgaris*	Neg
14	14c	Dark to light brown	Neg	Neg	Neg	IMViC	*Proteus sp.*	Neg
17	17a	Pink to red	Neg	Neg	Pos	IMViC	*E.coli*	Pos
22	22a	Bluish green to brownish	Neg	Neg	Pos	IMViC	*Enterobacter aerogenes*	Neg
24	24a	Pink to red	Neg	Neg	Pos	IMViC	*E.coli*	Pos
26	26a	Pink to red	Neg	Neg	Neg	IMViC	*Proteus mirabilis*	Pos
26	26b	Dark to light brown	Neg	Neg	Neg	IMViC	*Proteus mirabilis*	Pos
27	27a	Pink to red	Neg	Neg	Pos	IMViC	*E.coli*	Pos

number (No); negative (Neg); positive (Pos); MacConkey agar (MAC); identification (ID).

Table S2. *cont.*

Sample (No)	Isolate (No)	Colour on ChromID ESBL	Gram	Oxidase	MAC	Identification technique	ID	MDDST
28	28a	Pink to red	Neg	Neg	Pos	IMViC	*E.coli*	Pos
31	31b	Pink to red	Neg	Neg	Pos	API 20E	*Escherichia vulneris*	Pos
33	33a.1.	Pink to red	Neg	Neg	Neg	IMViC	*Proteus mirabilis*	Neg
33	33a.2.	Pink to red	Neg	Neg	Pos	API 20E	*E.coli*	Pos
33	33c	Pink to red	Neg	Neg	Neg	IMViC	*Proteus mirabilis*	Neg
33	33b	Dark to light brown	Neg	Neg	Pos	API 20E	*E.coli*	Pos
34	34a	Dark to light brown	Neg	Neg	Neg	IMViC	*Proteus mirabilis*	Neg
34	34b	Pink to red	Neg	Neg	Neg	API 20E	*Proteus mirabilis*	Neg
37	37a	Bluish green to brownish	Neg	Neg	Neg	API 20E	*Klebsiella pneumoniae*	Pos
37	37b	Pink to red	Neg	Neg	Pos	IMViC	*Enterobacter aerogenes*	Neg
38	38a	Dark to light brown	Neg	Neg	Pos	API 20E	*E.coli*	Pos
38	38b.1.	Pink to red	Neg	Neg	Neg	IMViC	*Proteus mirabilis*	Neg
38	38b.2.	Pink to red	Neg	Neg	Neg	IMViC	*Proteus mirabilis*	Neg
38	38c	Pink to red	Neg	Neg	Neg	IMViC	*Proteus mirabilis*	Neg
39	39a	Pink to red	Neg	Neg	Neg	API 20E	*Proteus penneri*	Neg
39	39c	Pink to red	Neg	Neg	Pos	IMViC	*E.coli*	Pos
39	39b	Bluish green to brownish	Neg	Neg	Neg	API 20E	*Proteus penneri*	Neg
39	39d	Bluish green to brownish	Neg	Neg	Neg	API 20E	*Enterobacter cloacae*	Pos
44	44a	Dark to light brown	Neg	Neg	Pos	IMViC	*E.coli*	Pos
46	46a	Pink to red	Neg	Neg	Pos	API 20E	*E.coli*	Pos
46	46c	Pink to red	Neg	Neg	Pos	API 20E	*Escherichia vulneris*	Pos
48	48a	Pink to red	Neg	Neg	Pos	IMViC	*E.coli*	Pos
49	49a	Pink to red	Neg	Neg	Pos	IMViC	*E.coli*	Pos

number (No); negative (Neg); positive (Pos); MacConkey agar (MAC); identification (ID).

Table S2. *cont.*

Sample (No)	Isolate (No)	Colour on ChromID ESBL	Gram	Oxidase	MAC	Identification technique	ID	MDDST
50	50a	Bluish green to brownish	Neg	Neg	Pos	IMViC	*Enterobacter aerogenes*	Neg
59	59a	Pink to red	Neg	Neg	Pos	IMViC	*E.coli*	Pos
66	66b.1.	Bluish green to brownish	Neg	Neg	Pos	IMViC	*Klebsiella pneumoniae*	Pos
66	66b.2.	Bluish green to brownish	Neg	Neg	Pos	IMViC	*Klebsiella pneumoniae*	Pos
66	66c.1.	Pink to red	Neg	Neg	Pos	IMViC	*Klebsiella pneumoniae*	Pos
66	66c.2.	Pink to red	Neg	Neg	Neg	IMViC	*Proteus mirabilis*	Neg
68	68b.1.	Pink to red	Neg	Neg	Pos	IMViC	*E.coli*	Pos
75	75a	Dark to light brown	Neg	Neg	Neg	IMViC	*Proteus* sp.	Pos
83	83a	Pink to red	Neg	Neg	Pos	IMViC	*E.coli*	Pos
83	83c	Bluish green to brownish	Neg	Neg	Pos	IMViC	*E.coli*	Pos
86	86a	Pink to red	Neg	Neg	Pos	IMViC	*E.coli*	Pos
86	86b	Dark to light brown	Neg	Neg	Pos	API 20E	*E.coli*	Pos
86	86c.1.	Dark to light brown	Neg	Neg	Neg	API 20E	*Hafnia alvei*	Neg
88	88a	Pink to red	Neg	Neg	Pos	IMViC	*E.coli*	Pos
89	89a.1.	Pink to red	Neg	Neg	Pos	IMViC	*E.coli*	Pos
91	91a	Pink to red	Neg	Neg	Pos	IMViC	*E.coli*	Pos
91	91b.1.	Pink to red	Neg	Neg	Pos	IMViC	*E.coli*	Pos
94	94a.1.	Pink to red	Neg	Neg	Neg	IMViC	*Morganella morganii*	Pos
94	94a.2.	Pink to red	Neg	Neg	Neg	IMViC	*Proteus mirabilis*	Neg
94	94a.3.	Pink to red	Neg	Neg	Pos	IMViC	*E.coli*	Pos
94	94a.4.	Pink to red	Neg	Neg	Neg	IMViC	*Morganella morganii*	Pos
94	94a.5.	Pink to red	Neg	Neg	Neg	IMViC	*Morganella morganii*	Pos

number (No); negative (Neg); positive (Pos); MacConkey agar (MAC); identification (ID).

Table S2. *cont.*

Sample (No)	Isolate (No)	Colour on ChromID ESBL	Gram	Oxidase	MAC	Identification technique	ID	MDDST
95	95a.1.	Pink to red	Neg	Neg	Pos	IMViC	*E.coli*	Pos
95	95b	dark to light brown	Neg	Neg	Pos	API 20E	*Escherichia vulneris*	Pos
95	95c.1.	dark to light brown	Neg	Neg	Neg	IMViC	*Proteus mirabilis*	Pos
95	95c.2.	dark to light brown	Neg	Neg	Neg	IMViC	*Proteus mirabilis*	Neg
98	98a.1.	Pink to red	Neg	Neg	Neg	IMViC	*Morganella morganii*	Neg
98	98b	bluish green to brownish	Neg	Neg	Pos	IMViC	*Enterobacter aerogenes*	Neg

number (No); negative (Neg); positive (Pos); MacConkey agar (MAC); identification (ID).
