# Supplementary material for: ESBL-Positive Enterobacteriaceae from Dogs of Santiago and Boa Vista Islands, Cape Verde: A Public Health Concern
Source: Antibiotics (Basel). 2023 Feb 23;12(3):447. doi: 10.3390/antibiotics12030447 (PMC10044620; doi:10.3390/antibiotics12030447)
Supplement: Supplementary file 1 [file antibiotics-12-00447-s001.zip › Table S3.pdf]

Supplementary file – 3

Table S3. Antimicrobial resistance and virulence profiles of the isolates under study.

ISOLATE (No)	LOCATION	SPECIES ID	RESISTANCE PROFILE I	R	MAR INDEX	VIRULENCE PROFILE LIP	Dnase	BF (h)	GEL	LEC	PT	HEM	V. INDEX
**1a**	BA	*Morganella morganii*	AMC 30*	CTX 30, CPT 30, AMP 10*, CN 10, DO 30*, ENR 5	0.57	-	+	48	-	-	-	γ	0.29
**5a**	BA	*E. coli*	CTX 30, CPT 30	AMP 10, DO 30	0.18	+	+	-	-	-	-	α	0.43
**5b**	BA	*E. coli*	CPT 30	CTX 30, AMP 10, DO 30	0.27	+	-	48	-	-	-	α	0.43
**5c**	BA	*E. coli*	CPT 30	CTX 30, AMP 10, DO 30, AMC 30	0.36	+	-	48	-	-	-	α	0.43
**5d**	BA	*E. coli*	-	CTX 30, CPT 30, AMP 10, DO 30	0.36	-	+	-	-	-	-	α	0.29
**10a**	BA	*Citrobacter freundii*	-	CTX 30, CPT 30, AMP 10*, CN 10, CIP 5, DO 30, ENR 5, AMC 30*	0.67	-	+	24	-	-	-	α	0.43
**10b**	BA	*Citrobacter freundii*	DO 30	CTX 30, CPT 30, AMP 10*, CN 10, CIP 5, ENR 5, AMC 30*	0.56	-	+	24	-	-	-	α	0.43
**10d**	BA	*Citrobacter freundii*	DO 30	CTX 30, CPT 30, AMP 10*, CN 10, CIP 5, ENR 5, AMC 30*	0.56	+	+	24	-	-	-	α	0.57
**13a**	BA	*E. coli*	CIP 5, ENR 5	CTX 30, CPT 30, AMP 10, CN 10, DO 30, AMC 30	0.55	+	-	-	-	-	-	β	0.29
**17a**	BA	*E. coli*	-	CTX 30, CPT 30, AMP 10, CIP 5, ENR 5, AMC 30	0.55	+	-	-	-	-	-	α	0.29
**24a**	BA	*E. coli*	CIP 5	CTX 30, CPT 30, AMP 10, CN 10, DO 30, ENR 5, AMC 30	0.64	+	-	-	-	-	-	α	0.29
**26a**	BA	*Proteus mirabilis*	-	CTX 30, CPT 30, AMP 10, CN 10, F 100*, DO 30*	0.44	+	+	-	+	-	+	γ	0.57
**26b**	BA	*Proteus mirabilis*	-	CTX 30, CPT 30, AMP 10, CN 10, F 100*, DO 30*	0.44	+	+	-	+	-	+	γ	0.57

*Intrinsic resistance; positive (+); negative (-); hours (h); cefotaxime (CTX); ceftaroline (CPT); ampicillin (AMP); amoxicillin/clavulanate (AMC); gentamicin (CN); doxycycline (DO); enrofloxacin (ENR); ciprofloxacin (CIP); nitrofurantoin (F); meropenem (MEM); intermediate (I); resistant (R); multiple antimicrobial resistance index (MAR INDEX); virulence index (V. INDEX) lipase (LIP); Deoxyribonuclease (DNase); biofilm (BF); gelatinase (GEL); lecithinase (LEC); protease (PT); haemolysins (HEM); alpha (α); gamma (γ); beta (β).

Table S3. *cont.*

ISOLATE (No)	LOCATION	SPECIES ID	RESISTANCE PROFILE		MAR INDEX	VIRULENCE PROFILE							V. INDEX
			I	R		LIP	Dnase	BF (h)	GEL	LEC	PT	HEM	
**27a**	BA	*E. coli*	ENR 5, AMC 30	CTX 30, CPT 30, AMP 10	0.27	+	-	48	-	-	+	α	0.57
**28a**	BA	*E. coli*	AMC 30	CTX 30, CPT 30, AMP 10, DO 30	0.36	+	-	-	-	-	+	γ	0.29
**31b**	BA	*Escherichia vulneris*	ENR 5	CTX 30, CPT 30, AMP 10	0.27	+	-	-	-	-	+	γ	0.29
**33a.2.**	BA	*E. coli*	ENR 5	CTX 30, CPT 30, AMP 10	0.27	+	-	-	-	-	+	γ	0.29
**33b**	BA	*E. coli*	ENR 5	CTX 30, CPT 30, AMP 10	0.27	+	-	-	-	-	+	γ	0.29
**37a**	BA	*Klebsiella pneumoniae*	CIP 5	CTX 30, CPT 30, AMP 10*, CN 10, F 100, DO 30, ENR 5, AMC 30	0.70	+	+	-	+	-	-	β	0.57
**38a**	BA	*E. coli*	-	CTX 30, CPT 30, AMP 10, ENR 5	0.36	+	-	-	-	-	+	γ	0.29
**39c**	BA	*E. coli*	AMC 30	CTX 30, CPT 30, AMP 10, CN 10, DO 30, ENR 5	0.55	+	-	48	-	-	+	α	0.57
**39d**	BA	*Enterobacter cloacae*	CIP 5	CTX 30, CPT 30, AMP 10*, ENR 5, AMC 30*	0.33	+	-	48	-	-	-	γ	0.29
**44a**	BA	*E. coli*	AMC 30	CTX 30, CPT 30, AMP 10	0.27	+	-	24	+	-	-	α	0.57
**46a**	BA	*E. coli*	CIP 5	CTX 30, CPT 30, AMP 10, DO 30, ENR 5	0.45	+	-	-	-	-	-	α	0.29
**46c**	BA	*Escherichia vulneris*	ENR 5	CTX 30, CPT 30, AMP 10	0.27	+	-	-	-	-	-	α	0.29
**48a**	BA	*E. coli*	CIP 5	CTX 30, CPT 30, AMP 10, ENR 5	0.36	+	-	24	-	-	-	α	0.43
**49a**	BA	*E. coli*	CIP 5, AMC 30	CTX 30, CPT 30, AMP 10, ENR 5	0.36	+	-	24	+	-	-	γ	0.43
**59a**	N	*E. coli*	DO 30	CTX 30, CPT 30, AMP 10, CIP 5, ENR 5	0.45	+	-	-	-	-	-	α	0.29
**66b.1.**	N	*Klebsiella pneumoniae*	CIP 5	CTX 30, CPT 30, AMP 10*, CN 10, F 100, ENR 5, AMC 30	0.60	+	-	24	+	-	-	α	0.57
**66b.2.**	N	*Klebsiella pneumoniae*	ENR 5	CTX 30, CPT 30, AMP 10*, CN 10, F 100, CIP 5, AMC 30	0.60	+	-	24	-	-	-	α	0.43

*Intrinsic resistance; positive (+); negative (-); hours (h); cefotaxime (CTX); ceftaroline (CPT); ampicillin (AMP); amoxicillin/clavulanate (AMC); gentamicin (CN); doxycycline (DO); enrofloxacin (ENR); ciprofloxacin (CIP); nitrofurantoin (F); meropenem (MEM); intermediate (I); resistant (R); multiple antimicrobial resistance index (MAR INDEX); virulence index (V. INDEX) lipase (LIP); Deoxyribonuclease (DNase); biofilm (BF); gelatinase (GEL); lecithinase (LEC); protease (PT); haemolysins (HEM); alpha (α); gamma (γ); beta (β).

Table S3. *cont.*

ISOLATE (No)	LOCATION	SPECIES ID	RESISTANCE PROFILE		MAR INDEX	VIRULENCE PROFILE							V. INDEX
			I	R		LIP	Dnase	BF (h)	GEL	LEC	PT	HEM	
**66c.1.**	N	*Klebsiella pneumoniae*	-	CTX 30, CPT 30, AMP 10*, CN 10, CIP 5, DO 30, ENR 5, AMC 30	0.70	+	-	24	+	-	-	α	0.57
**68b.1.**	N	*E. coli*	ENR 5, AMC 30	CTX 30, CPT 30, AMP 10, CN 10, CIP 5, DO 30	0.55	+	+	24	-	-	-	α	0.57
**75a**	N	*Proteus* sp.	DO 30	CTX 30, CPT 30, AMP 10, F 100	0.33	+	-	24	+	-	+	α	0.71
**83a**	N	*E. coli*	-	CTX 30, CPT 30, AMP 10, CIP 5, ENR 5	0.45	+	+	24	-	-	-	α	0.57
**83c**	N	*E. coli*	-	CTX 30, CPT 30, AMP 10, CIP 5, ENR 5	0.45	+	+	24	-	-	-	α	0.57
**86a**	N	*E. coli*	ENR 5	CTX 30, CPT 30, AMP 10, DO 30, AMC 30	0.45	+	+	24	-	-	-	β	0.57
**86b**	N	*E. coli*	ENR 5	CTX 30, CPT 30, AMP 10	0.27	+	-	-	-	-	-	α	0.29
**88a**	N	*E. coli*	CN 10, CIP 5, ENR 5	CTX 30, CPT 30, AMP 10	0.27	+	+	24	-	-	-	α	0.57
**89a.1.**	N	*E. coli*	ENR 5, AMC 30	CTX 30, CPT 30, AMP 10, DO 30	0.36	+	+	72	-	-	-	α	0.57
**91a**	N	*E. coli*	-	CTX 30, CPT 30, AMP 10, CIP 5, DO 30, ENR 5, AMC 30	0.64	+	+	-	-	-	-	α	0.43
**91b.1.**	N	*E. coli*	-	CTX 30, CPT 30, AMP 10, CIP 5, DO 30, ENR 5, AMC 30	0.64	+	+	48	-	-	-	α	0.57
**94a.1.**	N	*Morganella morganii*	-	CTX 30, CPT 30, AMP 10*, CN 10, DO 30*, ENR 5	0.57	+	-	24	+	-	-	β	0.57
**94a.3.**	N	*E. coli*	-	CTX 30, CPT 30, AMP 10, CN 10, DO 30, ENR 5	0.55	+	-	24	+	-	-	β	0.57

*Intrinsic resistance; positive (+); negative (-); hours (h); cefotaxime (CTX); ceftaroline (CPT); ampicillin (AMP); amoxicillin/clavulanate (AMC); gentamicin (CN); doxycycline (DO); enrofloxacin (ENR); ciprofloxacin (CIP); nitrofurantoin (F); meropenem (MEM); intermediate (I); resistant (R); multiple antimicrobial resistance index (MAR INDEX); virulence index (V. INDEX) lipase (LIP); Deoxyribonuclease (DNase); biofilm (BF); gelatinase (GEL); lecithinase (LEC); protease (PT); haemolysins (HEM); alpha (α); gamma (γ); beta (β).

Table S3. cont.

ISOLATE (No)	LOCATION	SPECIES ID	RESISTANCE PROFILE		MAR INDEX	VIRULENCE PROFILE							V. INDEX
			I	R		LIP	Dnase	BF (h)	GEL	LEC	PT	HEM	
**94a.4.**	N	*Morganella morganii*	-	CTX 30, CPT 30, AMP 10*, CN 10, DO 30*, ENR 5	0.57	+	-	24	-	-	-	β	0.43
**94a.5.**	N	*Morganella morganii*	-	CTX 30, CPT 30, AMP 10*, CN 10, DO 30*, ENR 5	0.57	+	-	24	+	-	-	β	0.57
**95a.1.**	N	*E. coli*	AMC 30	CTX 30, CPT 30, AMP 10, CIP 5, DO 30, ENR 5	0.55	+	-	-	-	-	-	α	0.29
**95b**	N	*Escherichia vulneris*	ENR 5	CTX 30, CPT 30, AMP 10	0.27	+	-	-	-	-	-	α	0.29
**95c.1.**	N	*Proteus mirabilis*	-	CTX 30, CPT 30, AMP 10, F 100*, DO 30*	0.33	+	+	-	+	-	+	γ	0.57

*Intrinsic resistance; positive (+); negative (-); hours (h); cefotaxime (CTX); ceftaroline (CPT); ampicillin (AMP); amoxicillin/clavulanate (AMC); gentamicin (CN); doxycycline (DO); enrofloxacin (ENR); ciprofloxacin (CIP); nitrofurantoin (F); meropenem (MEM); intermediate (I); resistant (R); multiple antimicrobial resistance index (MAR INDEX); virulence index (V. INDEX) lipase (LIP); Deoxyribonuclease (DNase); biofilm (BF); gelatinase (GEL); lecithinase (LEC); protease (PT); haemolysins (HEM); alpha (α); gamma (γ); beta (β).
